# Automated offline adaptive radiotherapy through CBCT imaging: A multidimensional feasibility study of hypersight and iterative CBCT

**DOI:** 10.1002/acm2.70853

**Published:** 2026-10-22

**Authors:** Ben R. Archibald‐Heeren, Aidan Leong, Nigel Middlebrook, Peter Phung, Sarah Hauville, Yunfei Hu, Gayan Singappulige, Trent J Aland

**Affiliations:** ^1^ Icon Group, Clinical Care Brisbane Queensland Australia; ^2^ Icon Cancer Centre Bowen Wellington New Zealand; ^3^ Icon Cancer Centre Holmesglen Moorabbin Victoria Australia; ^4^ Icon Cancer Centre Gosford Gosford New South Wales Australia

**Keywords:** Adaptive Radiotherapy, Automated Contouring, CBCT, Deformable Registration, HyperSight

## Abstract

**Background:**

Offline adaptive radiotherapy (OffART) offers potential dosimetric and clinical advantages by adjusting planned dosimetry in response to systematic patient changes throughout treatment, however implementation is associated with an increased labor burden. Technological innovations in automated contouring, image registration and on‐board imaging present the opportunity for an automated workflow that achieves the benefit of OffART with a reduced impact on staff workload.

**Purpose:**

To assess if the image quality of a ring‐based cone beam computed tomography (CBCT) imaging system is sufficient to enable automated contouring, deformable image registration (DIR), and dose calculation, thereby facilitating the implementation of an automated offline adaptive workflow.

**Methods:**

The treatment images for 40 clinical patients were retrospectively analyzed. The cohort spanned across multiple disease sites, including breast, thorax, abdomen, and male pelvis. Patients were selected from a clinical department utilizing the Varian Halcyon HyperSight (HypS) imaging system, and another department utilizing the Varian TrueBeam iterative cone‐beam CT (TB‐iCBCT) imaging system. Automated processes, without human intervention, were completed using the IGRT images and compared with planning simulation CT (pCT) images. Images were assessed for region of interest truncation, automated contouring agreement, DIR accuracy, and dose calculation accuracy using qualitative visual assessment, dice similarity coefficient (DSC) contour agreement and gamma analysis for dose calculation.

**Results:**

HypS imaging demonstrated improvements in reduced structure truncation with a reduction of 8% of structures with major or complete truncation. 13/21 organs demonstrated improved automated contouring consistency with pCT in HypS relative to TB‐iCBCT. Overall DSC results for DIR propagated contours were higher in the HypS cohort, partially due to TB‐iCBCT image truncation and partially due to improved registrations on the HypS images. HypS imaging demonstrated stronger agreement with pCT reported tissue density in heterogeneous patient anatomy than TB‐iCBCT imaging. Dose calculation gamma results for HypS were on average higher than TB‐iCBCT when comparing to the pCT calculated dose, however patient specific anatomical changes resulted in variability in results. Both imaging systems scored high gamma passing rates for calculations on pelvis cases.

**Conclusion:**

Improved image quality and field of view on the HypS imaging system results in improved system performance for several of the processes within the workflow for offline adaptation. Dose calculation and tissue density representation is of sufficient accuracy to warrant use of the imaging for offline adaptive radiotherapy. Automated contouring and contour propagation accuracy improved with HypS in some scenarios but remains the major barrier to a truly automated offline adaptive workflow not requiring human intervention.

## 1 INTRODUCTION

Adaptive radiotherapy (ART) has evolved from concept^1^ to clinical reality over the past decade, with published evidence supporting its advantages over conventional approaches.^2^, ^3^, ^4^, ^5^, ^6^ ART can be considered within three timeframes: offline, online, and inline.^7^ Performed over the course of treatment, offline ART (OFFART) addresses systematic changes in patient anatomy or position over the treatment course. Online ART (OART) addresses changes per fraction immediately prior to treatment delivery, thus accounting for both systematic and random errors. Inline or real‐time ART (INART) adjusts the plan live during treatment, actively accounting for intra‐fraction motion.^7^

Whilst both OART and INART require specific delivery systems, OFFART has the potential to be implemented without dedicated systems.^8^ Current literature has demonstrated the potential of OFFART in head and neck,^9^, ^10^ thoracic^11^, ^12^, ^13^ and pelvic diseases,^14^, ^15^ providing dosimetry advantages over the course of treatment.

Despite potential clinical advantages, the adoption of OFFART is hampered by unresolved challenges.^16^ The OFFART process requires a number of steps, including evaluation of patient geometry, determination of clinical relevance, acquisition of a new planning dataset reflecting the change, re‐contouring and re‐planning, quality assurance (QA) of the new plan, and rescheduling to account for the new treatment. The NRG Oncology's 2021 state‐of‐the‐art review^17^ identified the labor burden of these processes, along with training requirements as the major barriers to implementation of ART. Within these processes, the acquisition of a new planning dataset and the associated recontouring task are resource‐intensive and may result in delays to the replanning timeline. These delays can result in further anatomical changes that may make the replan no longer suitable.

Modern image guided radiotherapy (IGRT) utilizes 3D imaging to ensure accurate patient position, most commonly in the form of cone‐beam computed tomography (CBCT).^18^ Notably, the use of these images for an OFFART process, rather than a repeated planning simulation computed tomography (pCT), presents an opportunity for efficiency improvements and, on this basis, earlier corrections to patient dosimetry in the event of a significant variation. CBCT imaging suffers from a number of limitations that hamper its use in the full OFFART process,^19^ namely Hounsfield Unit (HU) reported density instability in varying scatter conditions, image truncation (due to field of view limitations) and poorer contrast to noise ratio in comparison to pCT imaging.^20^, ^21^ Despite evidence that dose calculations on CBCT images can be quite robust to minor imaging issues, iterative reconstruction algorithms on standard CBCT imaging is still limited to a dose accuracy within 2%–3% of CT calculations.^21^, ^22^ Beyond dose calculation, image quality degradation may also result in issues in deformable image registration (DIR),^23^, ^24^ automated contour generation/propagation,^2^ and calculations in heterogeneous conditions.^21^

In 2023, a new treatment imaging system, HyperSight (HypS) (Varian Medical Systems, Palo Alto, US), was clinically released. Utilizing new imaging hardware and reconstruction algorithms,^25^ early evidence has highlighted the potential of HypS in overcoming existing CBCT limitations. Early clinical evidence by Robar et al.^26^ demonstrated that HypS achieves Hounsfield unit accuracy within 15 HU of pCT for muscle, fat, bone, and lung tissues, enabling direct dose calculation on CBCT images. Subsequent commissioning, patient and phantom studies^27^, ^28^, ^29^, ^30^, ^31^, ^32^, ^33^ demonstrated image quality approaching that of conventional fan beam CT. American College of Radiology (ACR) benchmark testing^34^ confirmed the majority of HypS protocols pass all CT accreditation standards. The HypS imaging system incorporates a larger imaging panel which provides an increased field of view (FoV) compared to traditional CBCT platforms. These image quality improvements have potential ramifications for efficiency of ART processes. Improved HU stability, particularly in heterogeneous anatomy, has allowed for dose calculation accuracy within 1% of pCT imaging for a range of anatomical sites.^27^, ^29^ Improved soft tissue visualization in the pelvis, abdomen thorax and head and neck enable potential improvements in automated contouring on HypS datasets, though results remain organ specific.^35^, ^36^

The current research suggests the possibility that an automated offline adaptive solution utilizing the HypS imaging technology. To the authors knowledge, although individual components of this workflow have been investigated in isolation, no study has yet evaluated the full process across HypS and TB‐iCBCT image quality against the following image quality/process requirements:

1. Is HypS/TB‐ICBCT image quality of sufficient quality to allow for unsupervised AI contouring with current CT trained models?
2. Is HypS/TB‐ICBCT image quality sufficient for high quality deformable registration across the pelvis, abdomen and thoracic anatomy?
3. Is HypS/TB‐ICBCT image quality sufficient for radiotherapy offline adaptation calculation and, where necessary, replanning?

By resolving the above questions this study attempts to quantify improvements in HypS imaging over current iterative cone beam CT (iCBCT) reconstruction algorithms with respect to the feasibility of an efficient automated OFFART workflow. Specifically, assessment of image quality, automated contouring consistency, DIR performance, and dose calculation on retrospective clinical datasets is conducted on both HypS and TrueBeam iCBCT imaging systems and compared to simulation images acquired on a pCT scanner.

## 2 METHODS

Data from 40 patients was collected retrospectively from two departments within our institution treating on a V3.0 Varian TrueBeam linac with iterative CBCT imaging (TB‐ICBCT), and a Varian Halcyon V4.0 linac with the HyperSight (HypS) panel and iCBCT Acuros reconstruction algorithm. Data accrual was performed in line with approved ethics submissions as per the institution's research committee. Prior to the patient imaging within this study, the imaging systems of both linacs were commissioned, and corresponding CT‐ED curves were established and verified using the CIRS 062 M electron density phantom (Computerized Imaging Reference Systems, Virginia, US).

A review of retrospective patients across both departments was performed for cases treated over a period of 3 months from December 2023 to March 2024. The full study inclusion/exclusion strategy is provided in Figure 1. Inclusion of patients was restricted to radiotherapy of the breast, prostate, lung, esophagus, liver, and stomach with online treatment registration using TB‐iCBCT (TrueBeam) or HypS (Halcyon). Breast cases within the study were imaged with patients in breath hold. Abdominal and lung cases were imaged and treated with patients performing free breathing and thus incur potential motion artifact on both imaging systems. Cases following major surgical intervention (e.g. mastectomy, prostatectomy) were excluded. Prescriptions inclusive of nodal irradiation were permitted within the patient cohort for breast and prostate radiotherapy cases. No staging or ECOG status was stipulated within the inclusion criteria for the study. Following imaging review of all potential cases, a further set of cases were removed where IGRT image extents were insufficient to cover a minimum of 50% of the regions of interest. This resulted in the inclusion of a total of 323 images from 40 patients, comprising 208 HypS images and 115 TB‐iCBCT images, as detailed in Figure 1. For each patient, CBCTs within the study period were retrospectively analyzed. A representative sample of CBCTs across the treatment course were selected for analysis. This ranged from 5–18 fractional images per patient dependent on course length.

**FIGURE 1:**
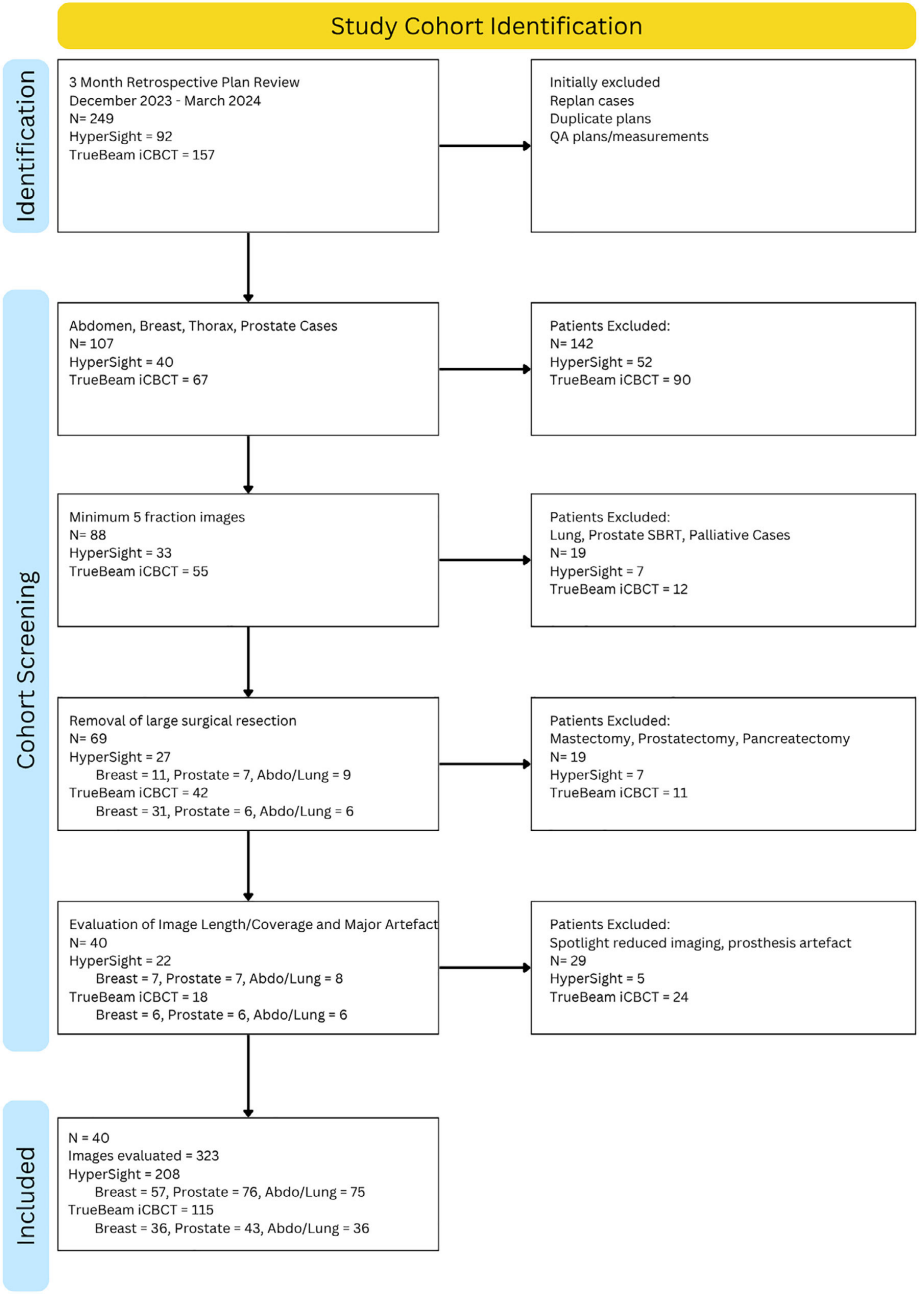
Study cohort inclusion/exclusion process.

## 3 AUTOMATED CONTOUR GENERATION

Automated contour generation was performed on all pCTs and analyzed treatment CBCT images across all patients. Contours were created using a commercial software, AutoContour v2.4 (Radformation, New York, USA) utilizing convolutional neural networks (CNNs) based on V‐Net architecture.^37^ The AutoContour algorithm was optimized for CT images, and no additional training of structure models was performed to improve contour accuracy on CBCT images.

Following contour generation on all datasets, evaluation of the automated contours was performed by clinical staff with at least 5 years of experience.

To assess feasibility of an automated adaptive trigger, no manual adjustment of the automated contours was permitted in the study. For each structure generated on the CBCTs, a visual assessment was performed to evaluate 1) the extent of the structure visible within the image (truncation assessment); and 2) agreement of the same structure generated between the CBCT and the pCT. Notably, the assessment was not of the accuracy of individual contours, but rather the consistency of contours generated between the patient CT and TB‐ICBCT/HS images, i.e., an anatomically incorrect contour would still be scored as matched if the anatomical error is consistent between datasets. The scoring criteria are listed in Table 1. Note that both Criterion 1 and 2 must be met for a given rating regarding contouring consistency (e.g. the contour must agree over 90% of the volume and maintain a surface within 5mm to be scored as “Matched”).

**TABLE 1:** Criterion for assessment of structure truncation and automated contour consistency between pCT and HypS/TB‐iCBCT images.

Truncation Assessment		
Score	Criteria	
None	Full structure is visible on the image	
Minor	70%‐99% of the structure is visible on the image	
Half	40%‐70% of the structure is visible on the image	
Major	10%‐30% of the structure is visible on the image	
Complete	<10% of the structure is visible on the image	

Automated Contouring Consistency		
Score	Criterion 1	Criterion 2
Matched	Contours agree over > 90% of the pCT contour	Maximum disagreement in contour anatomical edge is within 5 mm
Minor	Contours agree over > 75% of the pCT contour	Maximum disagreement in contour anatomical edge is within 10 mm
Major	Contours agree over > 50% of the pCT contour	Maximum disagreement in contour anatomical edge is within 25 mm
Failed	Contours agree < 50% of the pCT contour	Maximum disagreement in contour anatomical edge is over 25 mm
Uncontoured	No contour generated despite structure in image	

As a result of limited FoV, contours are sometimes truncated in CBCTs. Additionally, the amount of truncation may vary between CBCTs acquired across different fractions due to imaging settings and patient positioning. In this study, contours were evaluated only within the extent of the restricted image FoV. As such, a structure could be considered “matched” even when severely truncated on the CBCT as long as it meets the consistency criterion with the pCT within the CBCT bounds.

Following the above review, all contours that scored less than “Minor” for automated contour consistency were excluded from subsequent analyses. Additionally, only contours with minor or no truncation were included in the subsequent evaluation. Due to the length of the organ, spinal cord structures were included in analysis where truncation was scored as “Half” or greater. Lung cases were included for density analysis, but excluded from DIR evaluation, where truncation was scored as “Major” or above following quality confirmation of no truncation artifact. Whilst reducing the overall included data substantially for some organs this exclusion process was implemented to ensure data was collected from similar representations between the image sets.

### 3.1 Image registration evaluation

All CBCT images were rigidly registered to the pCT though their online registration used for treatment. The quality of this rigid registration was subsequently quantified by calculating the dice similarity coefficient (DSC) of automated contours.^38^ To minimize human intervention within the process, and to represent results of an automated solution, the DSC metric was scored without any correction for truncation of structures. Given the data curation for truncation defined above a lower bounds of DSC for accurate contour matching is expected to be 0.70 (excepting the spinal cord ROI).

In addition to the rigid registration, a DIR was performed between the CBCT and the pCT, followed by calculating the DSC. Improvements in DSC scores from the rigid registration to the DIR suggests a positional change of the structure relative to the daily online match and thus provides a potential trigger for ART.

The impact of auto‐contouring quality on this evaluation was controlled by limiting analysis to reviewed and approved contours (outlined in the prior section).

### 3.2 Image intensity and mass density stability

For all automated contours, average HU values were collected across all analyzed images. Through application of the previously commissioned CT physical density curves (CT‐PD) for all imaging systems, HU data was converted to mass density to allow for direct comparison between the image datasets.

### 3.3 Dose calculation

For all patients, a dose calculation was performed on the pCT and the CBCT (for both TB‐iCBCT and HypS cohorts) acquired during the first treatment fraction. The first fraction was selected in this analysis to minimize potential anatomical variations between the pCT and the CBCT. All calculations were performed with the v16.1 (HypS) or v15.6 (TB‐iCBCT) Eclipse Acuros algorithm (Varian Medical Systems, USA).

For each calculation, 3%/2 mm, 2%/2 mm and 1%/1 mm gamma analyses were performed between the dose calculations on the paired images using the pymedphys gamma analysis public library.^39^ Analysis was performed with full 3D gamma analysis following registration of the two distributions, restricted to the dose distribution present in the HypS/TB iCBCT image. A threshold of 20% used for the 3D analysis was used for all analysis. Gamma analysis was excluded from 15 mm from the patient surface to limit the impact of patient surface differences on the gamma analysis.

The mean percentage variation of dose across the target region was also recorded between paired images. The target region dose was determined by analyzing across data points at and above a 90% threshold of the intended prescription on each plan within the Eclipse treatment planning system. The geometrical size of these 90% threshold regions across the paired images was not controlled for in the analysis.

## 4 RESULTS

### 4.1 Data accrual and measurement

Across the 40 patients selected for the study, the auto‐contour algorithm was run on a total of 40 pCT and 323 CBCT datasets (208 HypS, 115 TB‐iCBCT), creating over 2,000 structures for analysis, including 19 distinct organ models across the abdomen, thorax, breast and male pelvis. Of the 15 breast cases that met the inclusion criteria, only a single HypS patient was right sided. Due to the impact of truncation and contour quality caused by isocenter offset the breast data is presented as ipsilateral and contralateral breast and lungs. A review of the right sided breast patient data determined this was not necessary for the remaining organs.

Results of truncation assessment and automated contouring consistency are provided in Figure 2.

**FIGURE 2:**
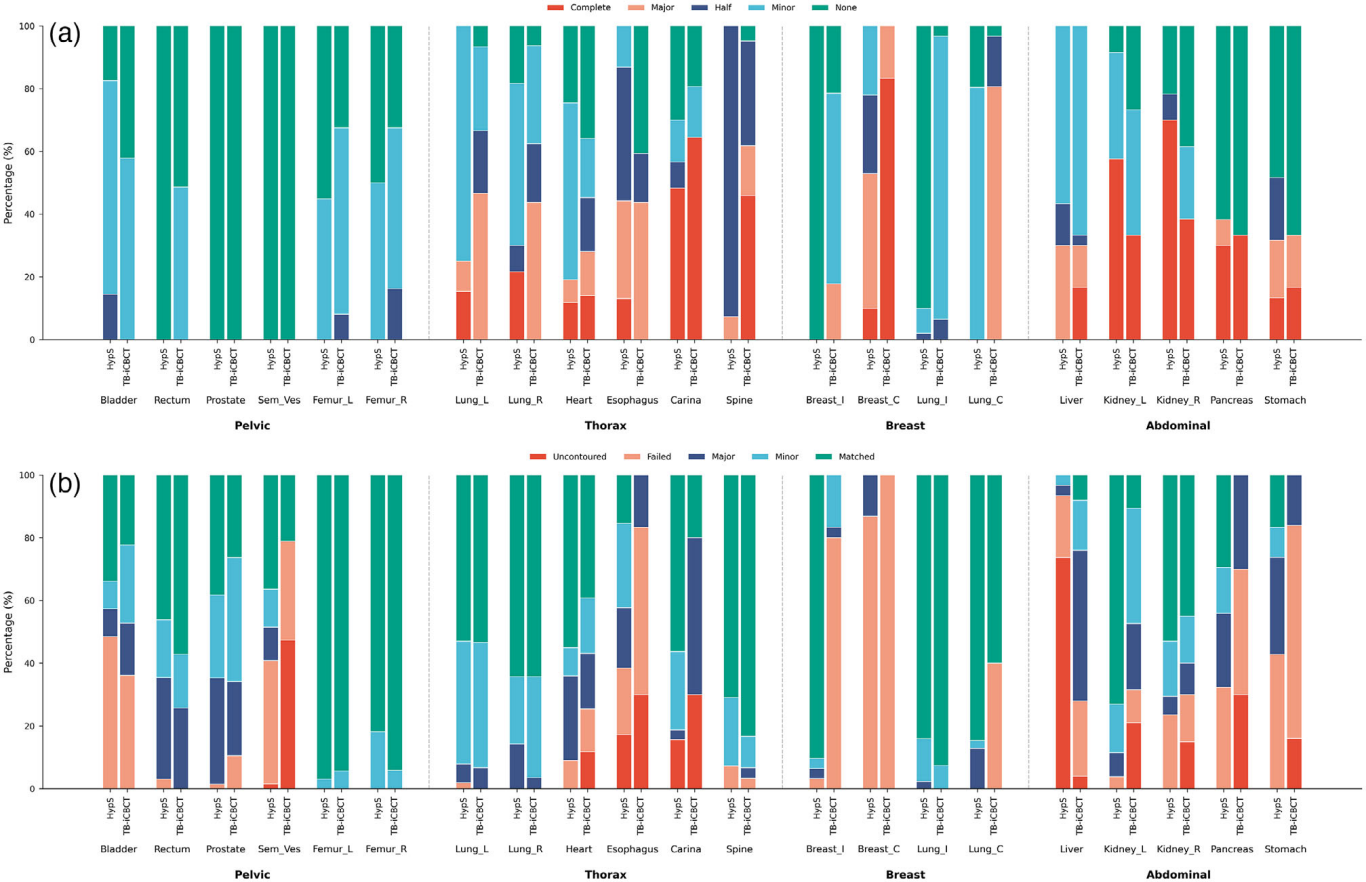
Qualitative assessment of (a) structure truncation and (b) automated contouring consistency between pCT and HypS/TB‐iCBCT images. N.B. Seminal Vesicles are abbreviated to Sem_Ves for clarity of the figure.

Overall truncation was reduced in the HypS cases with a reduction of 8% in the percentage of structures with major or complete truncation. The contralateral breast, contralateral lung, kidneys and spinal cord demonstrated the greatest levels of truncation across the dataset (Figure 2a). Structure truncation, on a per‐structure bases, was improved on HypS imaging apart from the bladder and kidneys.

Performance of the CT based automated contouring solution varied substantially across structures and anatomical sites on online image sets. HypS imaging resulted in 15/21 structures scoring a majority of contouring agreement with pCT automated contouring (“matched” or “minor”) in comparison to 12/21 for TB‐iCBCT). 13/21 structures resulted in improved qualitative contour assessments on HypS imaging compared to TB‐iCBCT. Greatest agreement with CT contours was achieved in high contrast structures such as the femurs and lungs for both image sets. The greatest variation in contouring performance between HypS and TB‐iCBCT was found for the Ipsilateral breast, kidneys, heart, stomach, pancreas and liver, with only the liver demonstrating superior performance on the TB‐iCBCT datasets.

The summary results of both deformable and rigid registrations are provided in Table 2, with extended data provided in Table S1. A restricted set of DSC results, where both HypS and TB‐iCBCT imaging resulted in a sufficient number of datasets for evaluation, are provided in Figure 3 for visual comparison. Results in the figure are split into abdomen (a), breast (b), thorax (c) and male pelvis (d). HypS imaging resulted in greater DSC metrics scores than TB‐iCBCT with the notable exceptions of the liver, Lung_R and seminal vesicles.

**TABLE 2:** DSC metric scores for all structures analyzed (meeting the truncation and contour similarity evaluation) following rigid and deformable (DIR) registration with the planning CT.

	Mean DSC								
	TB‐iCBCT			HypS			HypS‐ TB‐iCBCT		
Structure	Rigid	DIR	∆	RIG	DIR	∆	RIG	DIR	∆
**Abdomen**									
Esophagus	n/a	n/a	n/a	0.436	0.525	0.089	n/a	n/a	n/a
Liver^+^	0.761	**0.842**	0.081	0.601	0.657	0.088	−0.160	−0.185	0.007
Kidney_L	0.783	0.797	0.015	0.765	**0.848**	0.083	−0.018	0.051	0.068
Kidney_R	0.751	0.820	0.069	0.762	**0.882**	0.119	0.011	0.062	0.050
Stomach	n/a	n/a	n/a	0.467	**0.576**	0.109	n/a	n/a	n/a
Pancreas	n/a	n/a	n/a	0.411	**0.591**	0.180	n/a	n/a	n/a
Breast									
Breast_Ipsi	0.810	0.852	0.042	0.895	**0.939**	0.045	0.085	0.087	0.003
Breast_Contra	n/a	n/a	n/a	n/a	n/a	n/a	n/a	n/a	n/a
Lung_Ipsi	0.833	0.891	0.053	0.899	**0.962**	0.062	0.066	0.071	0.010
Lung_Contra	n/a	n/a	n/a	0.879	**0.952**	0.064	n/a	n/a	n/a
Heart	0.860	0.861	0.044	0.872	**0.960**	0.076	0.012	0.100	0.032
Spinal Cord	n/a	n/a	n/a	n/a	**0.197**	0.530	n/a	n/a	n/a
**Lung**									
Carina	0.750	0.838	0.088	0.755	**0.871**	0.113	0.005	0.033	0.025
Heart	0.808	0.830	0.021	0.865	**0.935**	0.071	0.057	0.106	0.050
Lung_L	0.896	**0.919**	0.022	0.850	**0.919**	0.058	−0.045	0.000	0.037
Lung_R	0.871	**0.932**	0.055	0.840	0.895	0.056	−0.031	−0.037	0.001
Spine	0.491	0.529	0.036	0.492	**0.645**	0.142	0.002	0.116	0.106
Male Pelvis									
Bladder	0.756	0.813	0.057	0.776	**0.824**	0.048	0.020	0.012	−0.008
HoF_L	0.738	0.804	0.066	0.769	**0.889**	0.120	0.031	0.084	0.053
HoF_R	0.735	0.791	0.056	0.828	**0.906**	0.078	0.093	0.115	0.022
Prostate	0.844	0.848	0.003	0.877	**0.894**	0.017	0.033	0.047	0.014
Rectum	0.754	0.774	0.020	0.731	**0.819**	0.089	−0.023	0.045	0.068
Seminal Vesicles	0.609	**0.692**	0.083	0.548	0.631	0.083	−0.061	−0.061	0.001

*Note*: Green color coding of the final columns demonstrates structures where HypS imaging resulted in an improved DIR performance, as determined by improvement in DSC, when compared to TB‐iCBCT. Red shows no improvement or degradation in HypS results relative to TB‐iCBCT. The highest average DSC scores for each structure across all images are denoted with bold text. Standard deviation of analysis is removed for readability, but is included in the full table within supplementary material S‐1.

^+^ Structure is heavily under sampled in HypS cohort (n < 5).

**FIGURE 3:**
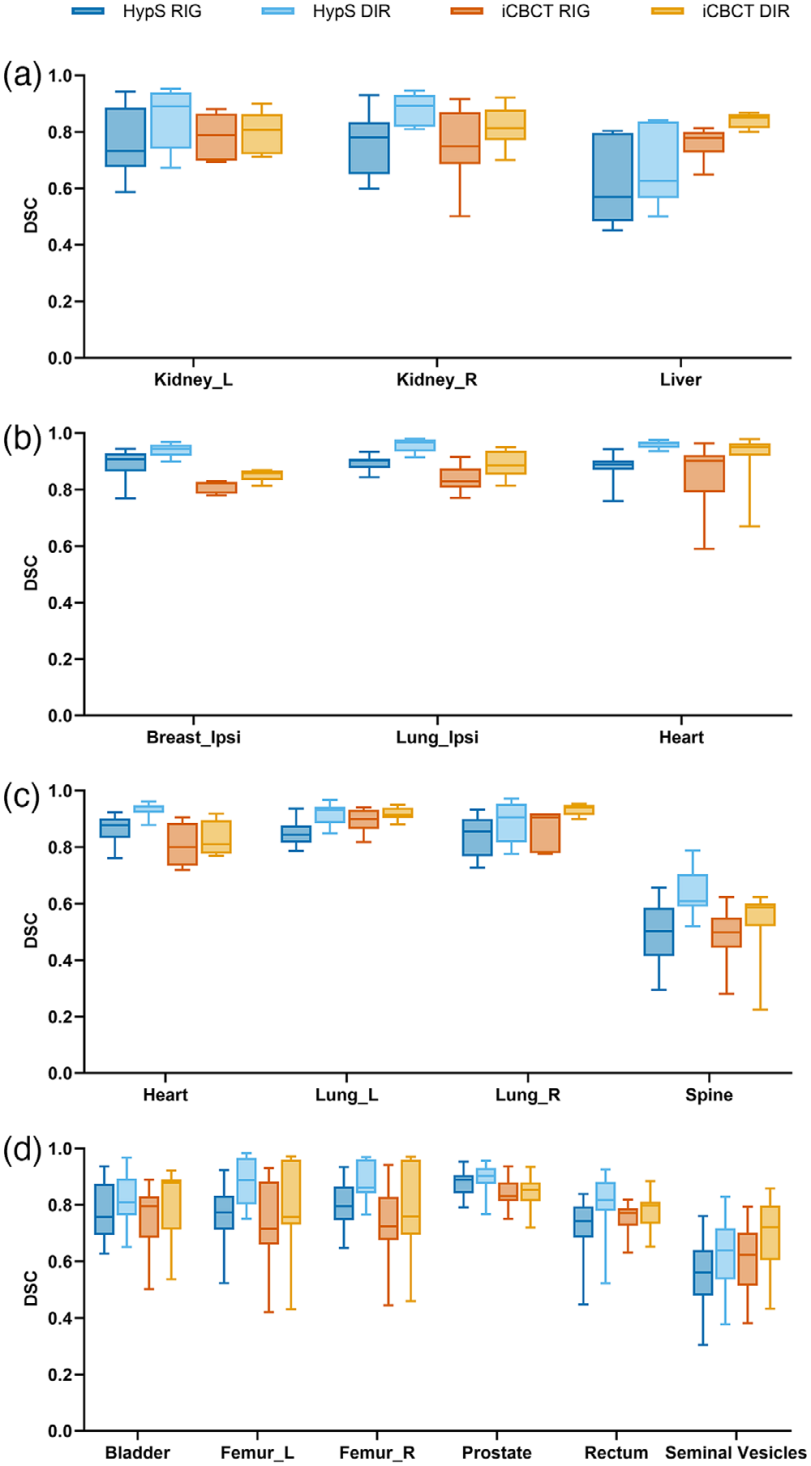
DSC distributions for rigid registration and DIR for HypS and TB‐iCBCT imaging in (a) abdominal, (b) breast, (c) thorax and (d) male pelvic cases. N.B. Results are not provided for either system for Breast_Contra, Pancreas, Stomach and Esophagus due to poor auto‐contouring performance in the TB‐iCBCT set. Liver data for the HypS cohort is provided in the plot but is under‐sampled (n < 5).

For all structures except the bladder HypS imaging resulted in greater DSC improvement from DIR registrations. Average DSC metric scores following DIR in the thorax and breast were 0.875 and 0.929 for TB‐iCBCT and HypS respectively. In the less heterogeneous anatomy (abdomen and pelvis) DIR DSC metrics fell to 0.798 and 0.817. Despite little to no truncation (Figure 2a) the seminal vesicles, pancreas and stomach demonstrated the worst DSC metrics.

Plots of CT‐PD derived density variation between online imaging and the pCT for each cohort are provided in Figure 4, supported by paired patient specific density variations between pCT and IGRT in Table 3 and extended analysis data in Table S2.

**FIGURE 4:**
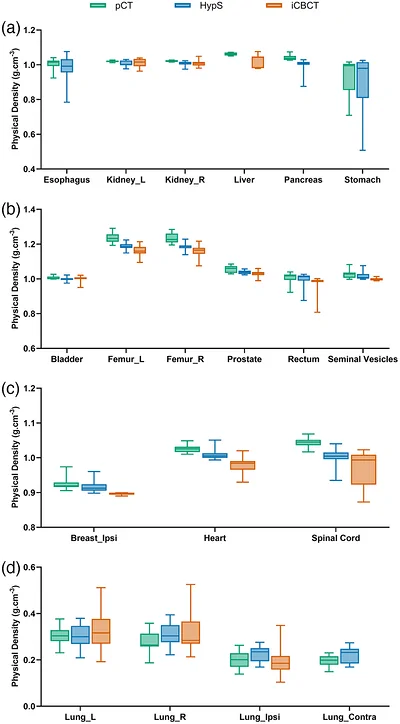
Image reported density for approved auto contoured structures between the pCT and two imaging study cohorts. Figure is split to improve interpretation‐ A) abdominal ROIs, B) Pelvis soft tissue and bone ROIs, C) breast soft tissue ROIs, D) Thoracic ROIs. Note that TB‐iCBCT results are not provided for Breast_Contra, pancreas, stomach and Esophagus due to poor auto‐contouring performance. No results are provided for the Liver in the HypS cohort due to under‐sampling following exclusion criteria but is reported in Table 3.

**TABLE 3:** Density variation between pCT and HypS and TB‐iCBCT imaging cohorts (calculated on a per patient basis) for reviewed and approved automated contour structures.

	Value ∆ from paired pCT Mean (Standard Deviation)							
	HypS				TB iCBCT			
Structure	HU		ρ (g.cm^−3^)		HU		ρ (g.cm^−3^)	
**Abdomen**								
Esophagus	−12.162	(0.0)	−0.022	(0.056)	N/A		N/A	
Kidney_L	−13.134	(17.9)	−0.018	(0.014)	−18.282	(35.6)	−0.023	(0.025)
Kidney_R	−9.314	(16.7)	−0.018	(0.013)	−15.561	(23.4)	−0.016	(0.017)
Liver^+^	46.166	(4.4)	0.019	(0.002)	−22.545	(43.0)	−0.032	(0.041)
Pancreas	−9.664	(15.9)	−0.018	(0.015)	N/A		N/A	
Stomach	23.193	(115.8)	0.018	(0.11)	N/A		N/A	
**Pelvis (Male)**								
Bladder	−11.666	(8.1)	−0.008	(0.008)	−1.261	(22.6)	0.000	(0.017)
Femur_L	−33.451	(72.5)	−0.054	(0.038)	−69.608	(76.1)	−0.062	(0.044)
Femur_R	−24.478	(65)	−0.045	(0.037)	−73.659	(78.1)	−0.064	(0.046)
Prostate	−2.722	(5.1)	−0.022	(0.012)	1.199	(22.5)	0.002	(0.025)
Rectum	−7.861	(64.5)	−0.013	(0.051)	−30.125	(56.5)	−0.022	(0.05)
Seminal Vesicles	−6.464	(9.4)	−0.020	(0.009)	−13.657	(12.4)	−0.009	(0.008)
**Thorax**								
Breast_Ipsi	−1.358	(18.7)	−0.013	(0.004)	−24.530	(1.7)	−0.020	(0.002)
Breast_Contra	N/A		N/A		N/A		N/A	
Carina	96.095	(322.1)	0.004	(0.058)	61.750	(1.3)	0.173	(0.001)
Heart	−15.193	(13.1)	−0.023	(0.009)	−44.325	(29.3)	−0.037	(0.02)
Lung_L	−26.151	(36.6)	0.019	(0.035)	11.009	(144.6)	0.095	(0.075)
Lung_R	−25.474	(28.0)	0.019	(0.027)	8.971	(91.6)	0.126	(0.073)
Lung_Ipsi (Breast)	−11.920	(124.5)	0.018	(0.015)	−26.912	(36.3)	0.014	(0.036)
Lung_Contra (Breast)	−14.097	(22.7)	0.032	(0.015)	−35.677	(12.1)	−0.002	0.015
SpinalCord	−35.039	(28.5)	−0.044	(0.019)	−47.410	(57.4)	−0.040	(0.052)

*Note*: Cell shading is provided to highlight results within ± 0.025cc (green), 0.05cc (orange) and outside 0.05cc (red). N.B. Absolute values are excluded from the table for readability, however, are included in full Table S2.

^+^ Liver data is provided but is heavily under sampled in the HypS cohort due to poor auto‐contouring and patient target location (< 5 organs included).

Overall average agreement with pCT reported density and on‐board imaging was ‐0.010 g.cm^−3^ ± 0.043 g.cm^−3^ for HypS imaging, compared with 0.005 g.cm^−3^ ± 0.116 g.cm^−3^ for TB‐iCBCT imaging. Overall HU variation, excluding the impact of system specific density curves, was ‐11.6HU ± 80.2HU and ‐28.9HU ± 73.3HU for HypS and TB‐iCBCT respectively. Density agreement with CT images was similar for both imaging solutions for the male pelvis cohort (HypS: ‐0.027 g.cm^−3^, TB‐iCBCT:‐0.026 g.cm^−3^). HypS imaging resulted in smaller variation from CT reported densities for thorax cases, including breast, (‐0.008 g.cm^−3^) in comparison to TB‐iCBCT imaging (0.028 g.cm^−3^). Overall variation in abdomen is not reported due to poor correlation in included contours passing review.

The gamma analysis agreement between CT calculations and fraction 1 calculations on HypS and TB‐iCBCT imaging is summarized in Table 4. Gamma analysis results across all images show clinically acceptable results at a 3%/2 mm criteria for both HypS and TB‐iCBCT imaging (99.5% ± 1.0% and 96.8% ± 2.9% respectively). Anatomical specific results demonstrate improved HypS performance in Breast and Prostate (improvement in 1%/1 mm gamma of 13.6% and 24.7%), equivalent results in lung (within ± 1%) and poorer gamma analysis results in abdomen cases (‐11.0% respectively). Calculations on the HypS imaging resulted in a greater percentage of the calculation dose covered within the image‐ on average 99.6% ± 1.0% for HypS compared with 90.9% ± 7.2% for TB‐iCBCT.

**TABLE 4:** Mean target dose calculation variation (comparing dose above the 90% threshold), gamma analysis and IGRT dose coverage results for first fraction calculations on each imaging cohort evaluated against the initial pCT.

				Dose Variation (%)		Average Passing Gamma Analysis (%)						Image Dose Cover (%)		
						1%/1 mm		2%/2 mm		3%/2 mm				
			*N*	Average	St Dev	Average	St Dev	Average	St Dev	Average	St Dev	Average	St Dev
Abdoment													
		HyperSight	4	1.4%	0.5%	85.4%	10.1%	96.1%	4.2%	98.6%	1.8%	100.0%	0.0%
		iCBCT	3	0.3%	0.3%	96.4%	3.2%	99.4%	0.6%	99.8%	0.4%	100.0%	0.0%
Breast													
		HyperSight	7	0.5%	0.6%	89.1%	4.0%	99.1%	0.6%	99.7%	0.2%	99.0%	1.6%
		iCBCT	6	1.3%	1.4%	75.5%	9.1%	92.0%	3.3%	95.2%	1.9%	89.4%	3.8%
Prostate													
		HyperSight	7	0.4%	0.6%	96.2%	2.6%	99.6%	0.5%	99.9%	0.1%	100.0%	0.0%
		iCBCT	6	−0.7%	0.6%	71.5%	19.0%	91.3%	6.1%	94.9%	3.1%	86.3%	6.7%
Lung													
		HyperSight	4	0.6%	1.6%	87.8%	11.7%	97.7%	1.7%	99.2%	1.2%	99.1%	1.4%
		iCBCT	4	0.2%	2.4%	87.4%	11.7%	98.2%	2.1%	99.1%	1.4%	99.1%	1.4%
													
All													
		HyperSight	22	0.7%	0.8%	90.5%	6.9%	98.5%	2.2%	99.5%	1.0%	99.7%	1.0%
		iCBCT	19	0.3%	1.2%	80.8%	15.4%	94.5%	5.2%	96.8%	2.9%	90.9%	7.2%

*Note*: N.B One TB‐iCBCT lung case included two plans, and as such incurs an extra plan analysis datapoint.

## 5 DISCUSSION

In this study, a multidimensional comparison was carried out among HypS, TB‐iCBCT, and pCT images, so as to assess the feasibility of an automated OFFART process based on HypS/TB‐iCBCT online imaging. The results revealed several advantages of HypS images over TB‐iCBCT images, demonstrating a clinical value.

### 5.1 Field of view impacts on daily imaging

HypS images demonstrate a FoV advantage in the adaptive calculation context. Across all assessed structure types, HypS incurred an 8% reduction in major or complete truncation when compared to TB‐iCBCT imaging. The greatest benefit was in breast cases, with the contralateral breast and contralateral lung reducing major/complete truncation by 59% and 50% respectively. Of the anatomical regions of interest reviewed in this study, only the spinal cord and the contralateral breast demonstrated consistent truncation with HypS imaging. Image truncation for the TB‐iCBCT imaging system was worst in breast patients due to patient offset, though truncation was also noted in central diseases of the abdomen and thorax due to the limited FoV length in the longitudinal axis. HypS imaging was still performed at the patient isocenter for breast cases, and these results reflect the opportunity the imaging system provides for ART of the breast.

### 5.2 Feasibility of using CT trained AI contouring on daily images

The larger FoV in HypS may provide a justification for the improvement in automated contouring consistency over the TB‐iCBCT imaging in some geometries. Poorer results on HypS aligned with organs truncated within the image set, either laterally (e.g. the right breast in left breast treatments) or in the longitudinal axis. Poor liver or kidney contouring results in this study are correlated with truncation of 20%–30% of the structure. HypS improvements in auto‐contouring relative to TB‐iCBCT acquisitions is demonstrated in heterogeneous anatomical sites in this work. HypS imaging allowed for all evaluated anatomical structures to be delineated at a level of minor or matched across at least one HypS image for all but the Breast_Contra structure. A total of 4 structures (Breast_Contra, Esophagus, Pancreas, Stomach) failed to achieve this rating across any TB‐iCBCT image. The result for the Breast_Contra structure is noteworthy‐ very poor automated contouring results are noted for even minor structure truncation. This is a limitation of CT trained CNN models and opens the possibility that a CBCT trained model, including truncated images, may be an avenue for development. Improvements in Esophagus, Pancreas and Stomach automated contouring align with improved low‐density structure visibility reported by Robar et al.^26^ Ipsilateral breast contouring performance was improved substantially in the HypS image set with consistent agreement in the HypS cohort in comparison to failure in the TB‐iCBCT cohort. This is a notable finding as it, along with the truncation and density accuracy results, signifies that HypS can support an offline adaptive breast program where TB‐iCBCT imaging fails.

Improvements in auto‐contouring results are not seen in the male pelvis cases, despite improvements in the image noise (Table 3). A subset of organs in the pelvis, such as the bladder, under‐performed with HypS against TB‐iCBCT images as demonstrated by a greater ratio of failed contours for HypS in Figure 2b. A comparison of pelvic artificial intelligence (AI) based contouring between HypS and iCBCT (TB‐iCBCT) by Sluijter et al.^40^ demonstrated no significant improvement achieved with the new imaging technology, however their work, which used a model trained on CBCT images and compared to manual contours, achieved overall superior results to those presented in this paper. Byrne et al.^41^ reported AI produced contours on previous iCBCT technology required minor or no edits in over 85% of fractions within a dedicated online adaptive system for a similar patient cohort. Recent work by Ashburner et al.^42^ demonstrated clinically acceptable AI contouring in over 80% of cases. All three papers utilized alternative AI models than those used in this study, highlighting the impact of AI modelling choices on contour results. All studies compared contours to manual experts rather than between CT and CBCT imaging implemented in this work. Results from Finnegan et al.^36^ mirror those of Byrne, Ashburner and Sluijter which suggest performance consistency between the imaging modalities and thus expect better results than those reported in this study.

Failure in these low contrast pelvic structures appears to be patient specific. A review of the failures in this study found no clear imaging artifact or truncation correlation between patients with excellent auto‐contouring results or complete failure. Failure in the bladder cases were not correlated with bladder filling. Where the AI contour failed this resulted in extension of the contour into the bowel region, resulting in very large contour errors and volume increases of over 100%. This represents individual examples where the use of AI contouring algorithms beyond their training set can result in major errors, and why careful evaluation of such algorithms is a necessity for clinical practice.

Overall results from Figure 2 demonstrate that for most structures, the introduction of HypS does not justify the unsupervised use of AI contouring for OFFART workflows with auto‐contour algorithms trained on CT datasets. Future improvements on models tailored to CBCT image quality, particularly images involving structure truncation, are required to improve overall performance.

### 5.3 Imaging impact on deformable registration

In cases where automated contouring performance was considered reasonable (either matched or with minor errors), substantial improvements in the DSC metric for the majority of ROIs are reported in HypS compared to TB‐iCBCT images (Figure 2, Tables 2 and S1). It should be noted that a component of this improvement in absolute DSC scores is a result of less structure truncation in HypS images due to its larger FoV. As analysis was performed against the pCT image, in cases where the original contour extends outside of the daily IGRT image, the DSC score will reduce.

Investigation of the relative change between rigid registration and DIR DSCs for the same images allows for some mitigation of the truncation effect. A review of the Table 2 demonstrates an increased improvement in DSCs between rigid registration and DIR in HypS over TB‐iCBCT imaging in all but the bladder structure. Whilst this may suggest image quality improvements in HypS imaging, resulting in superior DIR matching, the precise relationship between DIR accuracy and image quality, particularly image noise, is unclear within the literature and subject to specific algorithm implementations.^43^, ^44^, ^45^ Considerations should be given to the potential that this DSC improvement represents greater patient anatomical changes (particularly as the Halcyon linac in this study did not permit 6‐degrees of freedom corrections), and thus a greater metric increase from rigid to DIR, in the HypS cohort. For this consideration to hold, reduced rigid DSC scores would be expected in the HypS cohort (excluding the impact of truncation), which is not demonstrated in these results. To the authors awareness, this is the first validation of the impact of higher image quality from HypS on DIR accuracy.

As a workflow consideration, the change in the DSC metric between rigid registration and DIR may form as a potential adaptive trigger irrespective of ROI truncation. As the image is typically registered to ensure alignment with the treatment target, as is the intention of IGRT treatment process, the change in the DSC between the rigid and DIR registration is representative of the change in structure position from the original plan geometry relative to the target isocenter. A geometric example is provided below in Figure 5. Automatic tracking of a DSC delta/change metric may form a useful tool for automatically assessing change, irrespective of the overall completeness of the contoured organ. Clearly the above would not hold for dosimetry evaluation of organ at risk dose constraints and would be limited to anatomical changes.

**FIGURE 5:**
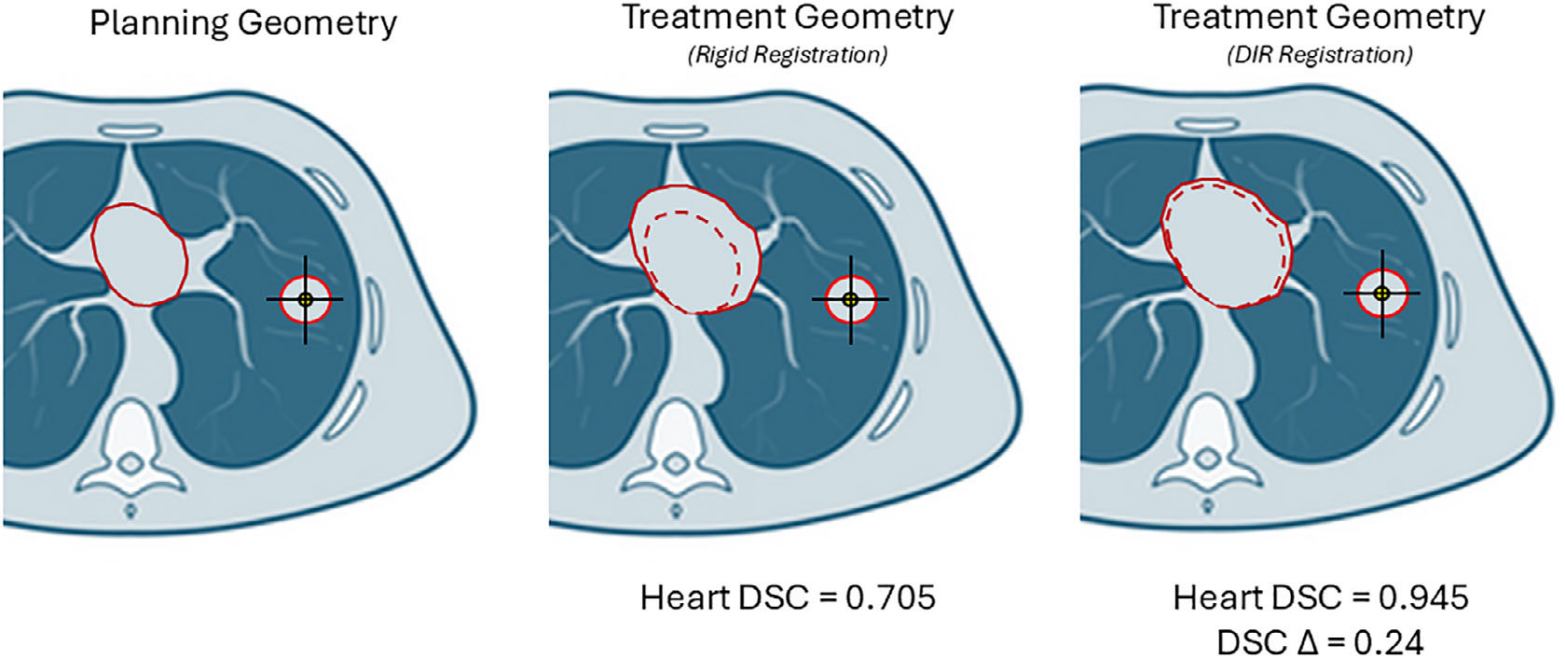
Rationale for the use of change in DSC between rigid and DIR registrations for tracking patient contour changes. The red contour represents the true organ geometry at treatment. The dotted line represents the rigidly (center) and deformably (right) propagated planning geometry. Note that final accuracy of the deformed structure is not required to allow for a geometry‐based trigger‐ only the relative improvement in DSC.

### 5.4 Density accuracy and variation

Results in Figure 4 and Tables 3 and S2 demonstrate an overall higher correlation of reported density to CT imaging for HypS in comparison to TB‐iCBCT imaging. When utilizing HU rather than density values, the agreement between the pCT and HypS images is in general agreement of findings of other studies (Sijtsema et al.^28^ (± 35 HU), Nelson^30^ (± 30HU) and Robar^26^ (± 15 HU). A notable result, as per Table 3 and Figure 4d, is that a strong HU agreement in lung did not result in a matched agreement in physical density, with a consistent offset of 0.02 g.cm^−3^. The authors institution undertook an extensive CT‐PD validation program prior to this research using multiple dedicated phantoms. That work determined, based on density plug agreement, that a HypS specific curve was required and was used in this work. Where assuming an approximate relationship of 0.001 g.cm^−3^ per HU in lung, and a very conservative 0.0007 g.cm^−3^ for tissue, correlation in density to CT in this study would be improved using the original planning CT curve, except for lung tissue despite the use of different kV between the pCT and HypS imaging (pCT: 120 kV, HypS: 125 kV). This study did not specifically control for patient breath‐hold levels, beyond standard treatment practice, and results in additional uncertainty for lung density results. These results may give additional weight to recommendations from existing literature that suggest individual HypS CT‐PD curves are not required.^26^

Table 3 demonstrates a general trend to reduced image noise and density structure variation in HypS over TB‐iCBCT imaging. The improvement in image quality supports previous findings from a number of image quality studies on the HypS system both with clinical datasets^26^, ^28^, ^32^ and phantom studies.^30^, ^31^, ^33^, ^34^ The improvement is most dramatic in heterogeneous regions in the thorax where the Acuros algorithm is expected to account for scatter change effects. Structures along heterogeneous paths, such as the breast, heart and thoracic spinal cord, demonstrate improvements in mean correlation with CT imaging and tighter density distributions.

Despite improvement in density accuracy of solid tissue proximal to lung tissue the results for the lung organs provide a pattern increased density variability (though reduced in comparison to the TB‐iCBCT cohort) in addition to the previously noted density offset. A follow up investigation of the HypS patient cohort found that these cases included 3 patients where anatomical changes (including variations in breathing magnitude, fibrosis of lung tissue, and significant abdominal gas changes) resulted in real density changes in the overall structure when compared to the CT image.

A notable result is the substantial improvement in density stability of the ipsilateral lung and breast with CT images in breast treatment, cases where the imaging isocenter is not centered within the patient habitus, supporting the feasibility of daily IGRT imaging for breast adaptive radiotherapy on the HypS system. The contralateral lung density performed poorly in these cases, despite study control for clear truncation artefact. The consistency of the offset in the experimental data suggests a minor truncation to the contralateral anatomy in these cases that warrants a future investigation. When interpreting Figure 4 it should be noted that the TB‐iCBCT cohort had consistently lower density in pCT images than the HypS cohort. As the lung_ipsi and lung_contra cohorts for both arms are considered in the pCT box plots this results in a visual exaggeration of the variation in density for HypS (to increased density) and TB‐iCBCT (to decreased density) and readers are directed to Table 3 and the paired analysis.

Mixed results are noted for other structures under motion (aforementioned Lung, as well as the Stomach and Esophagus in Table 3 and Figure 4a,d) which suggest HypS imaging may suffer from motion artifact. Abdomen and thorax clinical cases did not implement breath‐hold in this study, and further investigations with regards to improved density accuracy with motion management is warranted.

### 5.5 Dose calculation on treatment images

Despite overall improvement in image quality in HypS images, statistical significance is not reported in this work on account of insufficient data size. Overall improvement in dose calculation across the cohort is demonstrated with 1%/1 mm criteria (11.1%), with minor improvements across 2%/2 mm (4.1%) and 3%/2 mm (2.8%) analysis, as per the results in Table 4. This may suggest that, with respect to insensitive megavoltage radiotherapy beams, the density variations reported for TB‐iCBCT ‐a worst case of ∼0.173 g.cm^−3^ for smaller structures (carina) and ∼ 0.126 g.cm^−3^ for larger structures (lung_r)—are dominated by patient position, anatomical and surface changes. This is contradicted by Liu et al.^46^ who suggests tighter agreement than achieved in this work should apply, however dose calculations in an admittedly small heterogeneous cohort within this study suggests truncation as the major factor for dose accuracy over density accuracy.

Whilst the average gamma agreement does trend higher in the HypS dose calculation cohort, variability in gamma analysis for HypS imaging in the abdomen, where TB‐iCBCT results outperformed HypS, and lung are noted. Variability in results is also impacted by the degradations in gamma passing rates due to surface contours‐ particularly in breast cases‐ despite techniques to reduce its impact. Sijtsema et al.^28^ reduced the impact of these effects by comparing to deformed synthetic CTs in the CBCT geometry. This solution was not implemented within this study due to a lack of suitable commissioned DIR algorithm. For the anatomical changes in two thorax and one abdominal case, where gas was introduced in the stomach/bowels, mass conserving solutions such as DIR may not be suitable.

Mean target dose variance within the target region (determined by an 90% dose threshold) were 0.7% ± 0.8% and 0.3% ± 1.2% for HypS and TB‐iCBCT respectively. TB‐iCBCT imaging was still found to be robust for dose calculation in most scenarios with an average gamma analysis result of 96.8% for 3%/2 mm criteria and 94.5% for 2%/2 mm. In contrast to the HypS patient cohort, the abdominal patients in the TB‐iCBCT arm demonstrated particularly homogenous anatomy, and calculations were in high agreement with CT.

Previous literature has demonstrated that improvements in reconstruction algorithms in lung anatomy can improve the accuracy of dose calculation,^21^, ^47^ however similar improvements are restricted to breast cases in this study. The gamma analysis results presented in the pelvis and breast align with current literature.^29^, ^31^, ^47^, ^48^, ^49^

Results in this study for lung dose calculations (for both mean dose variation and gamma analysis) are well colerated with Sijtsema et al.,^28^ who compared calculations to deformed synthetic CTs. Duan et al.^27^ also implemented comparison against synthetic CT calculations for breast and pelvic indications and reported similar 1%/1 mm gamma evaluations to this work, though with much tighter variation than reported in this work. Comparisons in this study are compared to a rigid registration with the planning CT which likely incurs some degradation from the results of Sijtsema and Duan. As previously discussed, results in this study are also impacted by real patient anatomy within the lung HypS cohort. These effects are primarily evident at the 1%/1 mm criteria but should be considered within interpretation of dosimetric volume histogram revision of doses on HyperSight plans.

### 5.6 Study limitations

The analysis in this study covers a large number of patients across a range of anatomical sites with the intent to determine if a “hands‐off” solution is possible for OFFART monitoring. In covering a large number of cases, some specific anatomies incur a reduced number of datapoints that restrict the statistical power of the study. As such, statistical significance is not reported within this work. The cohort size for specific anatomical sites and structures is particularly impactful where automated contouring was not suitable in the TB‐iCBCT cohort. This results in some analysis not including data for the TB‐iCBCT imaging.

This could have been resolved by manually contouring failing datasets within the study. As the intent of the study is to examine if the individual components for an automated approach exist with the current imaging technology, this process of data collection was intentionally rejected from the methodology. The inability of a specific imaging modality to provide any automated contours of value for a specific organ represents an important result of the research.

The study also uses automated contour generation with an algorithm developed for CT, rather than CBCT. This design decision was intentional to ensure results were generally applicable to the clinical community where CT automated contouring solutions are more widely adopted. Whilst CBCT based algorithms may improve accuracy/consistency of treatment image contours they are uncommon at the time of writing and typically employed in dedicated offline adaptive solutions.

Finally, all datasets used in this study were retrospective and collated from real treatment imaging. Both the TB‐iCBCT and HypS imaging solutions have the option to performed “extended” CBCT, where two images are taken offset in the superior/inferior direction and then combined to create a longer dataset. Given a considerable component of the advantage of the HypS system in this study is the extended natural FoV it is likely that extended CBCT imaging on the TB‐iCBCT would resolve some of these issues and was not evaluated in this work. In any case, such imaging would incur repeat imaging on set, incurring a significant impact on treatment time and risk of patient positional change. For this reason the HypS imaging and TB‐iCBCT imaging in this study is compared as like‐for‐like with a single image acquisition.

## 6 CONCLUSION

The introduction of the HypS platform for daily imaging has improved daily treatment image quality closer to that of CT simulation, opening up the possibility of an automated OFFART process.

This study examined the impact of improved image quality on various components of the OFFART process, including automated contour generation, image registration, image density stability, and dose calculation with regards to the feasibility of automated offline adaptive evaluation.

HypS imaging demonstrates clinically feasible accuracy in DIR performance, accurate representation of tissue density, and dose calculation. A trend to improvements in these metrics is reported for HypS over TB‐iCBCT imaging without demonstrating statistical significance.

Whilst HypS demonstrated improvements over TB‐iCBCT in automated contouring for most organs within the study, the primary barrier to an automated OFFART workflow remains accurate contour generation.

## AUTHOR CONTRIBUTIONS

Ben R. Archibald‐Heeren conceived of the presented study, performed measurements and data analysis and wrote the manuscript with contributions from all authors. Ben R. Archibald‐Heeren also implemented the changes in text related to reviewers feedback. Aidan Leong conceived of the presented study, performed measurements and data analysis. Aidan Leong drafted some sections of the manuscript and performed review of the initial manuscript and subsequent revisions. Nigel Middlebrook performed measurements and data analysis within the study. Nigel Middlebrook drafted some sections of the manuscript and performed review of the initial manuscript and subsequent revisions. Yunfei Hu performed measurements and data analysis within the study. Yunfei Hu drafted some sections of the manuscript and performed review of the initial manuscript and subsequent revisions. Peter Phung performed measurements and data analysis within the study. Peter Phung drafted some sections of the manuscript and performed review of the initial manuscript and subsequent revisions. Sarah Hauville performed measurements and data analysis within the study. Sarah Hauville drafted some sections of the manuscript and performed review of the initial manuscript and subsequent revisions. Gayan Singappulige performed measurements and data analysis within the study. Gayan Singappulige drafted some sections of the manuscript and performed review of the initial manuscript and subsequent revisions. Trent J. Aland conceived of the presented study, assisted with technology procurement and partial funding for the research. Trent J. Aland provided substantial manuscript review for both the initial and revision submissions and drafted some sections of the original manuscript. All authors were involved in the consideration of reviewers feedback and included in the decisions with regards to manuscript changes.

## FUNDING INFORMATION

The work presented in this study is in part financially supported by research agreements between Siemens‐Healthineers and Icon Cancer Centres.

## CONFLICT OF INTEREST STATEMENT

Ben R. Archibald‐Heeren, Sarah Hauville, Yunfei Hu and Peter Phung have previous honorariums from Siemens‐Healthineers for speaking engagements at international conferences not related to this work. The other authors declare no conflicts of interest.

## ETHICS STATEMENT

This study was approved under by the Icon Cancer Foundation Ethics Committee with external review by Belburry Human Research Ethics Committee (Approval Number: HREC_2023‐10‐1269‐A‐1).

## Supporting information



Supporting Information

## Data Availability

The data analyzed in this manuscript is maintained on Icon Group servers in a de‐identified manner, as per the requirement in the research ethics approval. Data used in this study is available on request of the corresponding author.
