# Supplementary material for: Automated offline adaptive radiotherapy through CBCT imaging: A multidimensional feasibility study of hypersight and iterative CBCT
Source: J Appl Clin Med Phys. 2026 Oct 22;27(11):e70853. doi: 10.1002/acm2.70853 (PMC13691426; doi:10.1002/acm2.70853)
Supplement: Supplementary file 1 [file ACM2-27-e70853-s001.docx]

Table S-1

|  |  | TB-iCBCT | | | | | | HypS | | | | | | HypS- Tb-iCBCT | | |
| --- | --- | --- | --- | --- | --- | --- | --- | --- | --- | --- | --- | --- | --- | --- | --- | --- |
|  |  | Rigid DSC | | DIR DSC | | DIR DSC ∆ | | Rigid DSC | | DIR DSC | | DIR DSC ∆ | | Rigid DSC | DIR DSC | DIR DSC ∆ |
| Anatomy | Structure | Mean | St Dev | Mean | St Dev | Mean | St Dev | Mean | St Dev | Mean | St Dev | Mean | St Dev |  |  |  |
| Abdomen |  |  |  |  |  |  |  |  |  |  |  |  |  |  |  |  |
|  | Esophagus | n/a | n/a | n/a | n/a | n/a | n/a | 0.436 | 0.116 | **0.525** | 0.102 | 0.089 | 0.089 | n/a | n/a | n/a |
|  | Liver | 0.761 | 0.059 | **0.842** | 0.027 | 0.081 | 0.047 | 0.601 | 0.144 | 0.657 | 0.132 | 0.088 | 0.096 | -0.160 | -0.185 | 0.007 |
|  | Kidney_L | 0.783 | 0.079 | 0.797 | 0.072 | 0.015 | 0.016 | 0.765 | 0.117 | **0.848** | 0.106 | 0.083 | 0.071 | -0.018 | 0.051 | 0.068 |
|  | Kidney_R | 0.751 | 0.122 | 0.820 | 0.071 | 0.069 | 0.063 | 0.762 | 0.111 | **0.882** | 0.056 | 0.119 | 0.066 | 0.011 | 0.062 | 0.050 |
|  | Stomach | n/a | n/a | n/a | n/a | n/a | n/a | 0.467 | 0.111 | **0.576** | 0.175 | 0.109 | 0.093 | n/a | n/a | n/a |
|  | Pancreas | n/a | n/a | n/a | n/a | n/a | n/a | 0.411 | 0.184 | **0.591** | 0.192 | 0.180 | 0.177 | n/a | n/a | n/a |
| Breast |  |  |  |  |  |  |  |  |  |  |  |  |  |  |  |  |
|  | Breast_Ipsi | 0.810 | 0.022 | 0.852 | 0.022 | 0.042 | 0.021 | 0.895 | 0.043 | **0.939** | 0.020 | 0.045 | 0.032 | 0.085 | 0.087 | 0.003 |
|  | Breast_Contra | n/a | n/a | n/a | n/a | n/a | n/a | n/a | n/a | n/a | n/a | n/a | n/a | n/a | n/a | n/a |
|  | Lung_Ipsi | 0.833 | 0.043 | 0.891 | 0.053 | 0.053 | 0.033 | 0.899 | 0.023 | **0.962** | 0.021 | 0.062 | 0.020 | 0.066 | 0.071 | 0.010 |
|  | Lung_Contra | n/a | n/a | n/a | n/a | n/a | n/a | 0.879 | 0.024 | **0.952** | 0.030 | 0.064 | 0.028 | n/a | n/a | n/a |
|  | Heart | 0.860 | 0.117 | 0.861 | 0.139 | 0.044 | 0.034 | 0.872 | 0.055 | **0.960** | 0.012 | 0.076 | 0.036 | 0.012 | 0.100 | 0.032 |
|  | Spinal Cord | n/a | n/a | n/a | n/a | n/a | n/a | n/a | n/a | **0.197** | 0.116 | 0.530 | 0.027 | n/a | n/a | n/a |
|  |  |  |  |  |  |  |  |  |  |  |  |  |  |  |  |  |
| Lung |  |  |  |  |  |  |  |  |  |  |  |  |  |  |  |  |
|  | Carina* | 0.750 | 0.011 | 0.838 | 0.018 | 0.088 | 0.007 | 0.755 | 0.076 | **0.871** | 0.028 | 0.113 | 0.077 | 0.005 | 0.033 | 0.025 |
|  | Heart | 0.808 | 0.074 | 0.830 | 0.062 | 0.021 | 0.017 | 0.865 | 0.049 | **0.935** | 0.022 | 0.071 | 0.053 | 0.057 | 0.106 | 0.050 |
|  | Lung_L | 0.896 | 0.042 | 0.919 | 0.023 | 0.022 | 0.030 | 0.850 | 0.042 | **0.919** | 0.032 | 0.058 | 0.040 | -0.045 | 0.000 | 0.037 |
|  | Lung_R | 0.871 | 0.065 | 0.932 | 0.019 | 0.055 | 0.044 | 0.840 | 0.066 | **0.895** | 0.067 | 0.056 | 0.022 | -0.031 | -0.037 | 0.001 |
|  | Spine | 0.491 | 0.084 | 0.529 | 0.124 | 0.036 | 0.080 | 0.492 | 0.103 | **0.645** | 0.072 | 0.142 | 0.106 | 0.002 | 0.116 | 0.106 |
|  |  |  |  |  |  |  |  |  |  |  |  |  |  |  |  |  |
| Male Pelvis |  |  |  |  |  |  |  |  |  |  |  |  |  |  |  |  |
|  | Bladder | 0.756 | 0.121 | 0.813 | 0.120 | 0.057 | 0.033 | 0.776 | 0.100 | **0.824** | 0.084 | 0.048 | 0.041 | 0.020 | 0.012 | -0.008 |
|  | HoF_L | 0.738 | 0.129 | 0.804 | 0.138 | 0.066 | 0.036 | 0.769 | 0.103 | **0.889** | 0.079 | 0.120 | 0.089 | 0.031 | 0.084 | 0.053 |
|  | HoF_R | 0.735 | 0.124 | 0.791 | 0.149 | 0.056 | 0.054 | 0.828 | 0.081 | **0.906** | 0.071 | 0.078 | 0.059 | 0.093 | 0.115 | 0.022 |
|  | Prostate | 0.844 | 0.048 | 0.848 | 0.053 | 0.003 | 0.029 | 0.877 | 0.045 | **0.894** | 0.048 | 0.017 | 0.046 | 0.033 | 0.047 | 0.014 |
|  | Rectum | 0.754 | 0.051 | 0.774 | 0.057 | 0.020 | 0.040 | 0.731 | 0.088 | **0.819** | 0.084 | 0.089 | 0.047 | -0.023 | 0.045 | 0.068 |
|  | Seminal Vesicles | 0.609 | 0.135 | **0.692** | 0.142 | 0.083 | 0.048 | 0.548 | 0.132 | 0.631 | 0.118 | 0.083 | 0.127 | -0.061 | -0.061 | 0.001 |

*Supplementary Table S-1: DSC metric scores for all structures analysed (meeting the truncation and contour similarity evaluation) following rigid and deformable (DIR) registration with the planning CT. Colour coding of the final columns demonstrates structures where HypS imaging resulted in an improved DIR performance, as determined by improvement in DSC, when compared to TB-iCBCT. The highest average DSC scores for each structure across all images are denoted with bold text.*

*Table S-2*

|  | CT | | | | HypS | | | | | |  |  | TB iCBCT | | | |
| --- | --- | --- | --- | --- | --- | --- | --- | --- | --- | --- | --- | --- | --- | --- | --- | --- |
|  | HU | | PD | | HU | | PD | | Paired ∆ from CT | | HU | | PD | | Paired ∆ from CT | |
| Structure |  |  |  |  |  |  |  |  |  |  |  |  |  |  |  |  |
| **Abdomen** | | | | | | | | | | | | | | | | |
| Esophagus | -3.3 | (34.7) | 1.006 | (0.03) | -20.9 | (76.9) | 0.982 | (0.069) | **-0.022** | (0.056) | N/A | N/A | N/A | N/A | N/A | N/A |
| Kidney_L | 14.7 | (9.1) | 1.019 | (0.011) | 5.2 | (19) | 1.008 | (0.014) | **-0.018** | (0.014) | 10.0 | (34.8) | 0.997 | (0.025) | -0.023 | (0.025) |
| Kidney_R | 15.2 | (7.6) | 1.019 | (0.009) | 7.0 | (17.7) | 1.008 | (0.013) | -0.018 | (0.013) | 7.5 | (21.1) | 1.008 | (0.017) | **-0.016** | (0.017) |
| Liver | 47.9 | (8.9) | 1.060 | (0.011) | 92.4 | (4.3) | 1.082 | (0.002) | **0.019** | (0.002) | 16.1 | (52.7) | 1.014 | (0.041) | -0.032 | (0.041) |
| Pancreas | 20.0 | (16.3) | 1.026 | (0.015) | 6.1 | (17.8) | 1.008 | (0.013) | **-0.018** | (0.015) | N/A | N/A | N/A | N/A | N/A | N/A |
| Stomach | -80.1 | (107.9) | 0.933 | (0.106) | -133.3 | (158.4) | 0.874 | (0.151) | **0.018** | (0.11) | N/A | N/A | N/A | N/A | N/A | N/A |
| **Pelvis (Male)** | | | | | | | | | | | | | | | | |
| Bladder | 3.9 | (8) | 1.006 | (0.01) | -7.2 | (12.4) | 0.999 | (0.009) | -0.008 | (0.008) | -3.1 | (20.9) | 0.999 | (0.015) | **0.000** | (0.017) |
| Femur_L | 349.7 | (45.5) | 1.235 | (0.028) | 326.8 | (32) | 1.187 | (0.016) | **-0.054** | (0.038) | 281.0 | (61.1) | 1.171 | (0.037) | -0.062 | (0.044) |
| Femur_R | 345.2 | (47) | 1.233 | (0.029) | 321.8 | (37.7) | 1.185 | (0.018) | **-0.045** | (0.037) | 281.5 | (66.3) | 1.172 | (0.039) | -0.064 | (0.046) |
| Prostate | 45.1 | (16.2) | 1.054 | (0.021) | 48.3 | (17.1) | 1.047 | (0.017) | -0.022 | (0.012) | 32.0 | (20.5) | 1.032 | (0.018) | **0.002** | (0.025) |
| Rectum | -5.3 | (39.4) | 1.004 | (0.033) | -12.1 | (46.5) | 0.986 | (0.054) | **-0.013** | (0.051) | -27.8 | (43.8) | 0.982 | (0.04) | -0.022 | (0.05) |
| Seminal Vesicles | 21.5 | (23.2) | 1.028 | (0.027) | 20.5 | (23.6) | 1.019 | (0.023) | -0.020 | (0.009) | -4.8 | (12.5) | 0.999 | (0.008) | **-0.009** | (0.008) |
| **Thorax** | | | | | | | | | | | | | | | | |
| Breast_Ipsi | -95.0 | (14.2) | 0.926 | (0.017) | -99.7 | (15.8) | 0.918 | (0.015) | **-0.013** | (0.004) | -120.1 | (1.7) | 0.898 | (0.002) | -0.020 | (0.002) |
| Breast_Contra | -96.1 | (18.7) | 0.924 | (0.021) | #DIV/0! | #DIV/0! | 0.896 | 0.012 | N/A | N/A | N/A | N/A | N/A | N/A | N/A | N/A |
| Carina | -772.7 | (51.9) | 0.221 | (0.053) | -771.4 | (39.3) | 0.262 | (0.039) | **0.004** | (0.058) | -696.8 | (1.3) | 0.328 | (0.001) | 0.173 | (0.001) |
| Heart | 19.8 | (6.7) | 1.025 | (0.009) | 7.6 | (10.5) | 1.008 | (0.009) | **-0.023** | (0.009) | -34.3 | (29) | 0.994 | (0.035) | -0.037 | (0.02) |
| Lung_L | -694.2 | (37.2) | 0.304 | (0.04) | -730.4 | (47.6) | 0.302 | (0.046) | **0.019** | (0.035) | -698.4 | (82) | 0.326 | (0.077) | 0.095 | (0.075) |
| Lung_R | -716.4 | (43.3) | 0.278 | (0.043) | -723.3 | (46.9) | 0.309 | (0.045) | **0.019** | (0.027) | -712.1 | (79.4) | 0.313 | (0.074) | 0.126 | (0.073) |
| Lung_Ipsi (Breast) | -777.9 | (29.4) | 0.200 | (0.035) | -807.9 | (31.4) | 0.226 | (0.032) | 0.018 | (0.015) | -830.4 | (64.2) | 0.197 | (0.065) | **0.014** | (0.036) |
| Lung_Contra (Breast) | -789.7 | (27.3) | 0.189 | (0.025) | -808.6 | (35.4) | 0.224 | (0.032) | **0.032** | (0.015) | -864.073 | (12.1) | 0.164 | 0.015 | -0.002 | 0.015 |
| SpinalCord | 32.1 | (7.9) | 1.040 | (0.013) | 1.0 | (24.6) | 1.005 | (0.018) | -0.044 | (0.019) | -24.1 | (60.8) | 0.985 | (0.052) | **-0.040** | (0.052) |

*Supplementary Table S-2: Density variation between CT and HS and TB-iCBCT imaging cohorts (calculated on a per patient basis) for reviewed and approved automated contour structures. Cell shading is provided to highlight results within +/- 0.025cc (green), 0.05cc (orange) and outside 0.05cc (red).*
